# Human Detection in UAV Thermal Imagery: Dataset Extension and Comparative Evaluation on Embedded Platforms

**DOI:** 10.3390/jimaging11120436

**Published:** 2025-12-09

**Authors:** Andrei-Alexandru Ulmămei, Taddeo D’Adamo, Costin-Emanuel Vasile, Radu Hobincu

**Affiliations:** 1 Department of Electronic Devices, Circuits and Architectures, National University of Science and Technology Politehnica Bucharest, 060042 Bucharest, Romania; alexandru.ulmamei@upb.ro (A.-A.U.); costin.vasile1003@upb.ro (C.-E.V.); 2 Computer Science Department, National Institute of Applied Sciences of Lyon, 69621 Lyon, France; taddeo.d-adamo@insa-lyon.fr

**Keywords:** unmanned aerial vehicle, drone, search and rescue, machine learning, human detection, dataset

## Abstract

Unmanned aerial vehicles (UAVs) equipped with thermal cameras are increasingly used in search and rescue (SAR) operations, where low visibility and small human footprints make detection a critical challenge. Existing datasets are mostly limited to urban or open-field scenarios, and our experiments show that models trained on such heterogeneous data achieve poor results. To address this gap, we collected and annotated thermal images in mountainous environments using a DJI M3T drone under clear daytime conditions. This mountain-specific set was integrated with ten existing sources to form an extensive benchmark of over 75,000 images. We then performed a comparative evaluation of object detection models (YOLOv8/9/10, RT-DETR) and semantic segmentation networks (U-Net variants), analyzing accuracy, inference speed, and energy consumption on an NVIDIA Jetson AGX Orin. Results demonstrate that human detection tasks can be accurately solved through both semantic segmentation and object detection, achieving 90% detection accuracy using segmentation models and 85% accuracy using the YOLOv8 X detection model in mountain scenarios. On the Jetson platform, segmentation achieves real-time performance with up to 27 FPS in FP16 mode. Our contributions are as follows: (i) the introduction of a new mountainous thermal image collection extending current benchmarks and (ii) a comprehensive evaluation of detection methods on embedded hardware for SAR applications.

## 1. Introduction

Unmanned aerial vehicles (UAVs) equipped with thermal cameras have become essential tools for search and rescue (SAR) operations, providing rapid situational awareness in environments where visibility is poor and ground access is difficult. In such missions, automatic human detection is critical: victims may appear as small, low-contrast thermal footprints within complex backgrounds such as forests, snowfields, or rocky terrain. While deep learning–based object detectors such as the YOLO family have achieved remarkable accuracy on visible-spectrum data, their application to thermal UAV imagery remains challenging due to scale variability, sensor noise, and the scarcity of annotated datasets representative of real SAR conditions.

Existing public datasets in the field, such as FLIR ADAS, BIRDSAI, RGBTDronePerson, and HIT-UAV, cover primarily urban, open-field, or controlled environments, with limited representation of mountainous or high-altitude rescue scenarios. Moreover, these datasets differ substantially in sensor type, altitude, resolution, and annotation format, making it challenging to train models that generalize across domains. As a result, even state-of-the-art detectors tend to perform well within individual datasets but fail to maintain accuracy when multiple sources are combined, a phenomenon known as domain shift.

To address this limitation, we introduce a new thermal image collection acquired in mountainous regions under clear daytime conditions using a DJI M3T drone. This dataset complements existing benchmarks by focusing on SAR-specific scenes where humans appear as small or partially occluded thermal targets. We integrate it with ten widely used datasets to form a comprehensive meta-dataset, designed to evaluate model robustness under highly heterogeneous conditions.

Building upon this resource, we conduct an extensive comparison of object detection (YOLOv8–11, RT-DETR) and semantic segmentation (U-Net variants) models. The study examines accuracy, inference speed, and energy consumption on an NVIDIA Jetson AGX Orin, providing insights into the feasibility of real-time embedded deployment. Our results show that while YOLO models perform strongly on average, their accuracy degrades significantly when testing specifically on a small object sub-dataset.

The main contributions of this work are as follows:

- A new mountain-oriented thermal dataset extending existing UAV infrared benchmarks for search and rescue applications.
- A comprehensive evaluation of state-of-the-art detection and segmentation models on both desktop and embedded hardware.

## 2. Related Work

This section reviews the newest approaches and work related to human detection in multi-spectral streams and visible and infrared datasets.

### 2.1. Detection Algorithms

In [1], the authors propose real-time UAV human detection and gesture recognition using YOLOV3-tiny. Their system includes a body-gesture recognition phase and a custom dataset of ten rescue gestures captured via an onboard UAV camera. The system achieves 99.80% accuracy for body gestures and 94.71% for hand gestures. The study also emphasizes power efficiency and GPU optimization. In [2], the authors aim to improve detection performance under varying conditions by using a modified YOLOv5, tested with the VisDrone dataset and an F11 4K PRO drone. In [3], a YOLOv5 model is optimized via transfer learning for small datasets and challenging conditions, combining TIR and RGB images. Zhao et al. [4] introduce Mixed YOLOv3-Lite, designed for low-spec, non-GPU systems using YOLO-LITE with added ResBlocks and parallel subnetworks. In [5], a background subtraction approach enhances dim pedestrian detection using high-boost filtering and adaptive local thresholding. In [6], Tsai et al. use CNNs for detecting people in various postures from IR images, localizing them with bounding boxes. YOLOv4 outperforms other deep learning models in [6], achieving 65 FPS on 640 × 480 images with  98% accuracy. In [7], Guettala et al. train YOLOv7 on ground-based TIR data to detect humans from various UAV angles, demonstrating its real-time effectiveness.

### 2.2. Domain Adaptation

In thermal-based applications, existing RGB datasets can be leveraged to enhance detection accuracy by applying domain adaptation. Domain adaptation is a technique used in machine learning applications to transfer knowledge from a source domain to a target domain (in our scenario, from RGB-trained models or datasets to thermal). Recent research efforts [8,9,10,11,12,13] have highlighted various domain adaptation frameworks, demonstrating both the importance and increasing popularity of this technique. For example, Kim et al. introduced a multi-spectral, unsupervised domain adaptation strategy for thermal image semantic segmentation in [8], along with a real-world RGB-Thermal semantic segmentation dataset comprising 950 manually annotated ground-truth labels in 19 classes. In [9], Vibashan et al. improved the conventional domain adaptation techniques by implementing a framework for meta-learning the initial condition of the detector, demonstrating improved mAP on two datasets targeting vehicle and pedestrian detection. Rauch et al. investigated a method for generating fast annotations for the thermal dataset by utilizing detections from visible images [10].

Recent advances in domain adaptation specifically address the RGB-to-thermal transfer challenge through several architectural innovations. Do et al. [14] introduced D3T (Distinctive Dual-Domain Teacher), which employs a zigzagging adaptation strategy that progressively bridges the RGB-thermal gap through dual-domain pseudo-labeling, achieving significant improvements over single-stage adaptation methods. Their approach addresses the larger domain shift between visible and thermal modalities compared to conventional visible-to-visible adaptation. Wang et al. [15] proposed a Progressive Domain Adaptation framework (PDAT) for thermal infrared tracking that transfers knowledge from large-scale labeled RGB datasets without requiring extensive TIR annotations, demonstrating that adversarial-based global and local adaptation modules can effectively reduce distribution discrepancies.

For semantic segmentation tasks, Gan et al. [12] developed a Multi-Domain Attention Network that performs unsupervised RGB-to-thermal adaptation by learning domain-invariant features through attention mechanisms, outperforming prior methods on classification benchmarks. Zhang et al. [16] further advanced the field with an Unbiased Granularity Alignment approach specifically designed for thermal object detection, addressing the biased discrimination and negative transfer issues that commonly arise when adapting across spectral domains. These methods collectively demonstrate that explicit domain adaptation strategies can substantially improve thermal detection performance, particularly when thermal training data is limited or when deploying models trained primarily on RGB datasets.

Despite these advances, domain adaptation remains computationally expensive during training and requires careful hyperparameter tuning. Moreover, adaptation performance varies significantly depending on the source–target domain similarity: adapting from urban RGB datasets to mountainous thermal scenarios may require different strategies than urban-to-urban thermal adaptation.

### 2.3. Multimodal Fusion

Multimodal fusion is the process of accurately overlaying two or more images captured in different spectral ranges (thermal and visible). This is typically achieved via image registration (the process of accurately aligning two or more images). Thermal-visible fusion represents an alternative to thermal-only detection methods, as it leverages information from both thermal and visible spectra. This comes with the disadvantage of being more computationally expensive, as thermal-visible fusion is a computationally intensive task. Wu and Liu introduced a technique for Region-of-Interest (RoI) extraction from virtual scenes in [17], which reduces the area to be registered, thereby effectively reducing the computation required. In the past decade, numerous research efforts have been made to enhance UAV-based thermal-visible registration. The majority of research articles were published in the last five years, indicating a growing interest in this subject [18,19,20,21,22,23]. Improving thermal-only detection methods (our work) will also enhance knowledge on multimodal fusion.

RGB-thermal fusion architectures can be categorized into three main paradigms: early fusion, intermediate (feature-level) fusion, and late (decision-level) fusion. Early fusion concatenates raw RGB and thermal inputs before feature extraction, offering simplicity and preservation of cross-modal correlations at the cost of increased computational complexity [24,25]. El Ahmar et al. [24] demonstrated that sigmoid-activated gating mechanisms for early fusion can improve detection performance by up to 9% by adaptively weighting modality contributions. Intermediate fusion combines features extracted separately from each modality, balancing computational efficiency with effectiveness for multi-defect detection tasks [25]. Late fusion operates on detection outputs from independent single-modal detectors, as demonstrated by Sousa et al. [26], who applied YOLOv5 separately to RGB and thermal streams before fusing predictions, achieving robust human detection in diverse lighting conditions.

Recent work has focused on adaptive fusion mechanisms that adjust modality weights based on environmental conditions. Wang et al. [27] introduced M2FNet, which improves low-light detection accuracy by 25.6% through multimodal feature fusion and identified eight illumination thresholds for optimal modal selection. For video object detection, Yuan et al. [28] proposed hybrid fusion with progressive interaction and temporal–modal difference modeling, addressing the unique challenges of fusing time-varying RGB-thermal video streams.

Representative RGB-thermal datasets that support fusion research include the KAIST Multi-spectral Pedestrian Dataset [29], CVC-14 for vehicle detection [30], FLIR ADAS for autonomous driving [31], and the City Scene RGB-Thermal MOT Dataset for multi-object tracking [24]; the RGBTDronePerson dataset [32] is among the few publicly available resources focusing on drone-based multi-spectrum person detection from high angles with complex backgrounds. The scarcity of well-registered aerial RGB-thermal datasets continues to limit research on multimodal fusion for UAV applications.

### 2.4. Synthetic Data Generation

Another potential way to mitigate the limitation of publicly available thermal datasets is to generate synthetic data. Creating completely synthetic data in the context of thermal imagery is unfeasible or extremely expensive due to the difficulty of modeling all the physical properties of thermal scenes [33]. The solution is to augment existing authentic images with synthetic objects, thereby transforming plain background images into scenes containing objects of interest. Madan et al. introduced a technique in [34] for 3D modeling and the insertion of objects into images containing only background. Qazi et al. leverage the existing visible datasets by proposing a method that estimates the thermal images from their visible image counterparts [35]. To enhance the quality of synthetic objects, Bianchi et al. proposed an augmentation pipeline that includes a stage for modeling and adding thermal noise to the synthetically generated objects [36]. Several other works available in the literature imply deep learning techniques for generating synthetic thermal images [33,37,38,39].

The effectiveness of synthetic thermal data depends critically on physical accuracy and domain characteristics. While synthetic augmentation has shown promise for improving model robustness under controlled scenarios [33,34], several caveats limit its applicability. First, physically accurate thermal rendering requires modeling complex heat transfer, material emissivity, atmospheric effects, and temporal thermal dynamics—factors that are often oversimplified in current pipelines. Second, synthetic data trained models may suffer from domain gap when tested on real-world imagery if the simulation parameters do not match deployment conditions (e.g., different times of day, weather, or altitude) [35]. Third, augmentation helps most when combined with real data in appropriate ratios; purely synthetic training typically underperforms hybrid approaches [37]. Recent work by Vo et al. [38] on generative models for synthetic thermal images shows that careful thermal noise modeling is essential to avoiding unrealistic signatures. Thus, synthetic data generation should be viewed as a complement to—not a replacement for—real-world data collection, particularly for safety-critical applications such as SAR where detection failures have serious consequences.

### 2.5. Embedded Inference

Recent progress in deep learning has enabled object detection models such as the YOLO family to achieve real-time performance on desktop GPUs. However, deploying these architectures on embedded systems remains a significant challenge due to strict limitations in computational resources, memory, and power consumption. As autonomous drones and edge devices increasingly perform onboard perception tasks, the need for efficient inference directly on embedded hardware has become critical. Table 1 highlights seven recent attempts on deploying YOLO models on NVIDIA-embedded GPUs. A broader overview of hardware acceleration paradigms, including GPU, FPGA, ASIC, and low-power accelerators, is comprehensively reviewed in [40].

Successful embedded deployment requires navigating multiple competing constraints: model accuracy, inference latency, memory footprint, energy consumption, and thermal management. Quantization represents the primary optimization strategy, converting floating-point weights and activations to lower-precision representations (FP16, INT8, or even INT4). As shown in Table 1 and our results (Section 4.1), FP16 quantization typically retains 99%+ of full-precision accuracy while approximately halving inference time and energy consumption. INT8 quantization offers further speedups (1.5–2× over FP16) but at the cost of 10–20% accuracy degradation, making it viable only when baseline performance has sufficient margin [41].

Beyond quantization, architectural choices significantly impact deployability. Light-weight variants such as EdgeYOLO [42] and EL-YOLO [43] achieve 34–44% mAP on VisDrone at 34 and 3 FPS, respectively, on Jetson Xavier, demonstrating that careful backbone simplification and decoupled detection heads can maintain acceptable accuracy while meeting real-time constraints. Model selection must also consider power budgets: EL-YOLO reports 9W average power consumption, making it suitable for battery-powered UAVs, whereas heavier models may require active cooling or shortened flight times. Memory bandwidth and on-chip cache utilization are additional bottlenecks; batched inference improves throughput but increases latency, which is problematic for real-time applications [44].

For SAR deployment, the tradeoff between detection sensitivity (recall) and processing speed is particularly acute. Missing a human target due to aggressive model compression or low frame rate sampling could have life-or-death consequences. Our work addresses this by systematically measuring accuracy–latency–energy Pareto fronts across multiple quantization levels and power modes (Section 4.2), providing practitioners with data-driven guidance for deployment decisions.

**Table 1:** Comparison of YOLO-based implementations on NVIDIA-embedded platforms.

Paper/Model	mAP	FPS	Dataset	Device	Quantization	Comments
EdgeYOLO [42]	44.8%	34	VisDrone	Jetson Xavier	FP16	Lightweight YOLO-style detector with decoupled head and simplified backbone for edge deployment.
Quantized Object Detection for Real-Time Inference on Embedded GPU Architectures (YOLOv4) [41]	71.36%	16	KITTI	Jetson AGX	FP32	Quantized YOLOv4 achieving real-time inference on low-cost Jetsons; minimal accuracy loss (<2%).
Quantized Object Detection for Real-Time Inference on Embedded GPU Architectures (YOLOv4) [41]	68.33%	47	KITTI	Jetson AGX	FP16	Quantized YOLOv4 achieving real-time inference on low-cost Jetsons; minimal accuracy loss (<2%).
Quantized Object Detection for Real-Time Inference on Embedded GPU Architectures (YOLOv4) [41]	56.69%	62	KITTI	Jetson AGX	INT8	Quantized YOLOv4 achieving real-time inference on low-cost Jetsons; minimal accuracy loss (<2%).
EL-YOLO [43]	44.1%	3	VisDrone	Jetson Xavier	FP16	9W average power consumption.
A Novel Smart System with Jetson Nano for Remote Insect Monitoring (YOLOv7) [45]	77.2%	5	Custom insect dataset	Jetson Nano	FP32	YOLOv7 applied for insect detection.
Counting People and Bicycles in Real Time Using YOLO on Jetson Nano (YOLOv5s) [46]	60.51%	10	Custom urban dataset	Jetson Nano	FP32	The paper evaluates multiple YOLOv5 variants.

Additionally, Lazarevich et al. conducted a comprehensive comparison of more than 500 YOLO-based object detection models, testing them on multiple datasets and four different hardware platforms (x86 CPU, ARM CPU, Nvidia GPU, and NPU) [47].

### 2.6. Datasets

FLIR ADAS [31] offers annotated thermal/visible frames for CNN-based detection. Though designed for driving, its paired images and person labels make it useful. VisDrone [48] is a large-scale UAV benchmark with 288 videos, 10,209 images, and 2.6 M annotated boxes. It features a diverse range of scenes, objects, and densities across 14 Chinese cities. It is split into 6471 training, 548 validation, and 3190 testing images. OTCBVS [49] is a comprehensive thermal/color dataset comprising 16 subsets. OSU Thermal [50] targets pedestrian detection using 284 thermal images (360 × 240, <30 Hz) from a rooftop-mounted Raytheon 300D sensor. OSU Color-Thermal [51] contains 17,089 color/thermal images (320 × 240, 30 Hz) from three scenes, using Raytheon PalmIR 250D and Sony TRV87 cameras. [32] introduces RGBTDronePerson and VTUAV-det for drone-based multi-spectrum detection. Captured from high angles with complex backgrounds, these datasets focus on the challenges of small objects. VTUAV-det adapts [22]’s tracking dataset for detection. BIRDSAI [52] is a challenging, low-quality thermal dataset comprising 61,994 nighttime aerial images of humans and animals, facilitating human–animal distinction. HIT-UAV [53] is a high-altitude IR dataset for UAV detection, with metadata like altitude, view angle, and daylight conditions. ETH-ASL’s Thermal IR Dataset [54] has 4381 annotated images (plus 2418 backgrounds) of humans, a cat, and a horse—useful for false positive filtering. LLVIP [55] is a low-light visible-IR dataset with annotations. The IR subset is valuable for aerial scenes with occlusions. [56] offers a thermal aerial dataset of forested areas, featuring people and dogs in occluded scenes. Only images are provided, with no annotations.

## 3. Methods

Our study evaluates object detection and semantic segmentation methods based on accuracy and inference speed. Precision, recall, and real-time performance are key metrics. Segmentation models are also tested for their pixel-level detection strengths. This section introduces the methods we used for dataset preparation, model training, and model evaluation.

### 3.1. Object Detection

The objective is to identify the most effective model for detecting people in thermal drone footage, with a primary focus on object detection models suitable for embedded systems.

#### 3.1.1. Dataset

Since no suitable data were available for search and rescue in mountainous environments, we collected a new set of images to support both this study and future research on thermal human detection. Our experiments used two resources: a collection of newly annotated images, and a larger meta-set combining material from ten different sources (including our mountain data).

Our mountain dataset comprises frames that were acquired with a DJI M3T drone under clear daytime conditions. Flights were performed at various altitudes and with varying camera inclinations, introducing substantial variation in target scale and viewpoint. All images were manually annotated for a single class (humans). This mountain-oriented material captures challenging conditions in which people frequently appear as small or partially occluded thermal footprints, providing a valuable complement to existing UAV infrared benchmarks that are mostly restricted to urban scenarios.

The meta-dataset combines multiple sources and newly annotated images to maximize diversity and completeness. To achieve this, images were taken from ten sources: BIRDSAI [52], RGBTDronePerson [32], VTUAV-det [32], FLIR ADAS V2 [31], HIT-UAV [53], ASL-TID [54], OTCBVS (Dataset01) [49], LLVIP [55], and Dataset thermal images of people-forested areas [56]. These datasets were identified in the literature as relevant to the study domain, and the project team acquired a custom one during mountain expeditions to serve the interests of the current research. The images were annotated using CVAT [57] and MakeSense.ai [58].

The following process was implemented to obtain the heterogeneous dataset:

- Performed a literature review and searched for thermal images datasets containing labeled instances of humans;
- Transformed all annotations to the YOLO format;
- Filtered the datasets to keep only thermal images (for datasets targeting both thermal and visible spectra);
- Filtered the annotations to keep only those targeting humans (‘human’, ‘people’, ‘pedestrian’, etc.);
- Renamed all resulting labels to ‘human’;
- The resulting annotations were manually checked to remove any potential outliers;
- Prepended the original dataset source name to the resulting image files for traceability;
- Added images from our own custom mountainous dataset;
- Balanced the dataset so every source contributes roughly equally to the model’s learning.

Datasets were cleaned, formatted, and balanced to include only thermal images and human annotations. All annotations were converted to YOLO format, which may have resulted in the loss of occlusion data. Outliers were checked and found to be minimal, with low risk of affecting results. Image counts vary by source, so dominant sources were selected to balance influence. Dataset names were prepended to filenames to preserve source traceability.

While combining heterogeneous datasets increases coverage and sample diversity, it also introduces substantial domain shifts (e.g., differences in sensor resolution, capture altitude, background context, and annotation styles). We did not apply domain adaptation or harmonization methods in this study, as our primary goal was to benchmark baseline performance across datasets. However, this limitation should be considered when interpreting the results.

**Figure 1:**
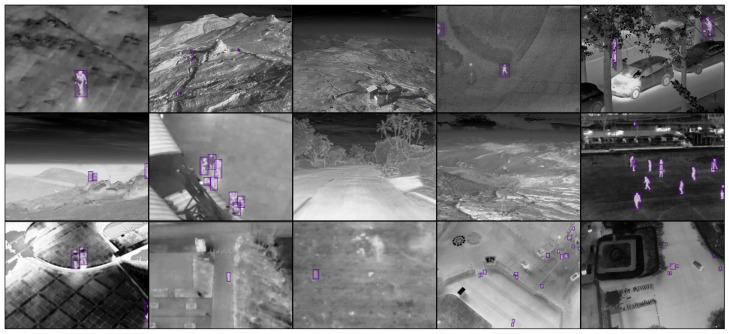
Mosaic preview of dataset images with purple detection boxes.

The final dataset comprises over 75,000 images (30% backgrounds) from ten varied sources. To request access to the database, please fill in the form at https://arh.dcae.pub.ro/projects/people-detection-systems-for-uav/thermal-aerial-images-database-for-people-detection/ (accessed on 25 August 2025). The split is 60/20/20 for training, validation, and testing, keeping source proportions balanced. Thermal images were resized to a 640-pixel width for model input, maintaining the aspect ratio. The average height after resizing is 458 pixels. Ground-truth bounding boxes are small: from 4 px^2^ (min) to 40 px^2^ at the 10th percentile. A mosaic preview of 15 images from the dataset is shown in Figure 1.

#### 3.1.2. Algorithms and Models

The YOLO models were selected for their state-of-the-art precision and speed. RT-DETR was included for comparison, given its reported real-time performance.

Six models were tested on the meta-dataset: YOLOv8 (M, X), YOLOv9 (E), YOLOv10 (M, X), and RT-DETR (L). YOLOv8 was chosen early on for its solid tradeoff between speed and accuracy. The M and X sizes were used to assess the cost–benefit of scaling.

At the study’s start, YOLOv8 was the latest stable release, with five COCO-pre-trained sizes. The M model was chosen for its high precision and lower inference time [59], and compared with the X variant to evaluate real-time feasibility and precision–cost tradeoffs. YOLOv8 was selected for its balance of speed and accuracy.

YOLOv9 was released in February 2024. It offers a higher $mAP_{50–95}$ and lower parameter/GFLOPS counts than YOLOv8, suggesting reduced inference costs. Only the largest pre-trained size (E) was used for comparison with YOLOv8 X.

YOLOv10 followed in May 2024, and while its declared $mAP_{50–95}$ is slightly lower than that of YOLOv9, it still surpasses YOLOv8 and improves latency and efficiency. Despite its late release, YOLOv10 (M, X) was included for comparison.

RT-DETR was tested only in the large version.

All six models were trained on the meta-dataset using an RTX 3090 (24 GB vRAM), with early stopping after 50 stagnant epochs and a maximum of 300 epochs.

Towards the end of the study, we also performed a limited exploratory test with YOLOv11 [59], the most recent Ultralytics release at the time of the experiment. This experiment was run only on the mountain-specific subset (7000 images, approximately 22,000 bounding box annotations) and is reported separately from the main YOLOv8–10 evaluation. While YOLOv11 is still an emerging implementation and not widely documented in the literature, we include it here to provide an early comparison.

#### 3.1.3. Embedded Platform

The models were deployed on a Jetson AGX Orin Developer Kit (64GB RAM, Ampere GPU). The system runs JetPack SDK 5.1.2, including Jetson Linux 35.4.1 (Kernel 5.10), Ubuntu 20.04, CUDA 11.4.19, cuDNN 8.60, and TensorRT 8.5.2. PyTorch 2.1.0, ONNX Runtime 1.16.1, and jetson-stats were also installed. Code execution used Python 3.8 and Ultralytics v8.2.28 [59].

#### 3.1.4. Evaluation

Evaluation focused on balancing detection accuracy, inference speed, and energy consumption across models and hardware. This required defining measurement methods for accuracy, latency, and power use. Optimizations were applied to ensure representative performance.

Precision (P), Recall (R), F1-score, Average Precision (AP), and Average Recall (AR) were used to evaluate detection performance. While AP, especially mAP, is the standard metric, AR was also emphasized due to the application context: in search and rescue, missing a human signal is more critical than a false positive, which the drone can verify. AR, introduced by Hosang et al. [60], is also widely used in COCO challenges. This study evaluates a single-class (human) detection problem, so AP and mAP, as well as AR and mAR, are equivalent. However, the task is multi-scale: object sizes vary significantly, with many instances being only a few pixels in area. To capture this, metrics were computed globally and across three size categories—small, medium, and large—based on bounding box area. The smallest and largest 30% of instances define the small and large groups, enabling scale-specific analysis.

#### 3.1.5. Statistical Evaluation

To assess the robustness and statistical significance of our results, each model configuration was trained five times with different random seeds (7, 42, 999, 2024, 12, 345) while keeping all other hyperparameters identical. For each run, we computed all evaluation metrics on the fixed test set. We report mean values with 95% confidence intervals obtained via the t-distribution and presented in Table 2. To test for statistically significant differences between models, we performed pairwise Wilcoxon signed-rank tests on the metric values across runs, applying Holm–Bonferroni correction for multiple comparisons. Differences are considered statistically significant at p<0.05 after correction. This approach enables us to distinguish meaningful performance differences from random variation due to weight initialization and training stochasticity.

#### 3.1.6. Embedded Costs

Inference costs were evaluated over the full test set by measuring latency (per-image processing time) and energy usage on the Jetson AGX Orin. Latency was obtained both from the Ultralytics Python package [59] and from independent timing of the full inference routine. Power consumption was monitored using the board’s five INA3221 channels, which track energy use across power rails [61]. Energy estimates followed a method inspired by the OpenDR jetson_power script [62], adapted for Orin by using jetson-stats for 30 Hz power sampling. Idle power was estimated via a 20 s pre-inference phase with 10 samples. Tests were run under two power configurations: maximum (MaXN, 60W with jetson_clocks enabled) and minimum (15 W with clocks disabled), as in [63]. PyTorch models were exported to ONNX and TensorRT formats, with TensorRT variants quantized to FP16 and INT8 (calibrated on the validation set) to assess performance–accuracy tradeoffs in embedded scenarios.

### 3.2. Semantic Segmentation

In drone-based search and rescue operations, individuals often appear small and occupy a minimal image space. Semantic segmentation offers pixel-level precision, making it more effective than traditional object detection for identifying such targets. To address this, we evaluated seven segmentation models originally developed for medical imaging. These include U-Net [64], known for its accuracy on small datasets, and its nested variant UNet++ [65], which uses dense skip connections for enhanced feature flow. Other models adapt U-Net with additional architectures: ResUNet combines it with ResNet [66]; Inception U-Net integrates multi-scale Inception modules [67]; and Dense U-Net incorporates DenseNet-style feature reuse [68]. Attention U-Net introduces attention gates to focus on relevant features [69], while SE U-Net uses squeeze-and-excitation blocks to model inter-channel dependencies [70].

## 4. Results

### 4.1. Accuracy

Different accuracy metrics were applied to accommodate the characteristics of each method.

#### 4.1.1. Object Detection

**Figure 2:**
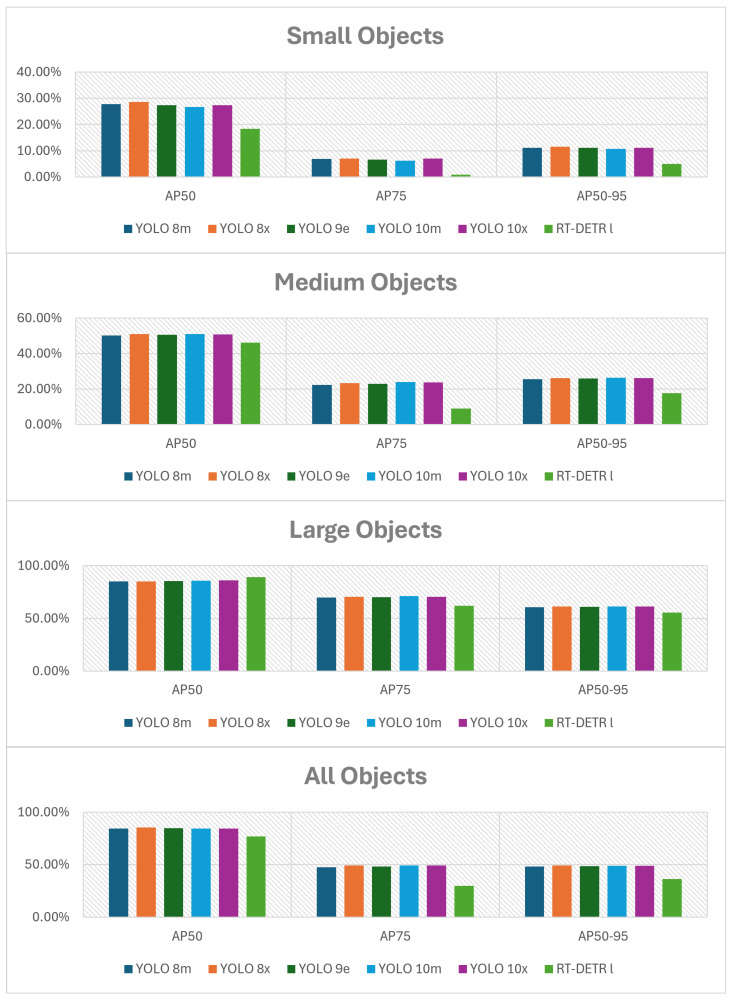
Comparison of AP metrics across different object sizes and models.

**Figure 3:**
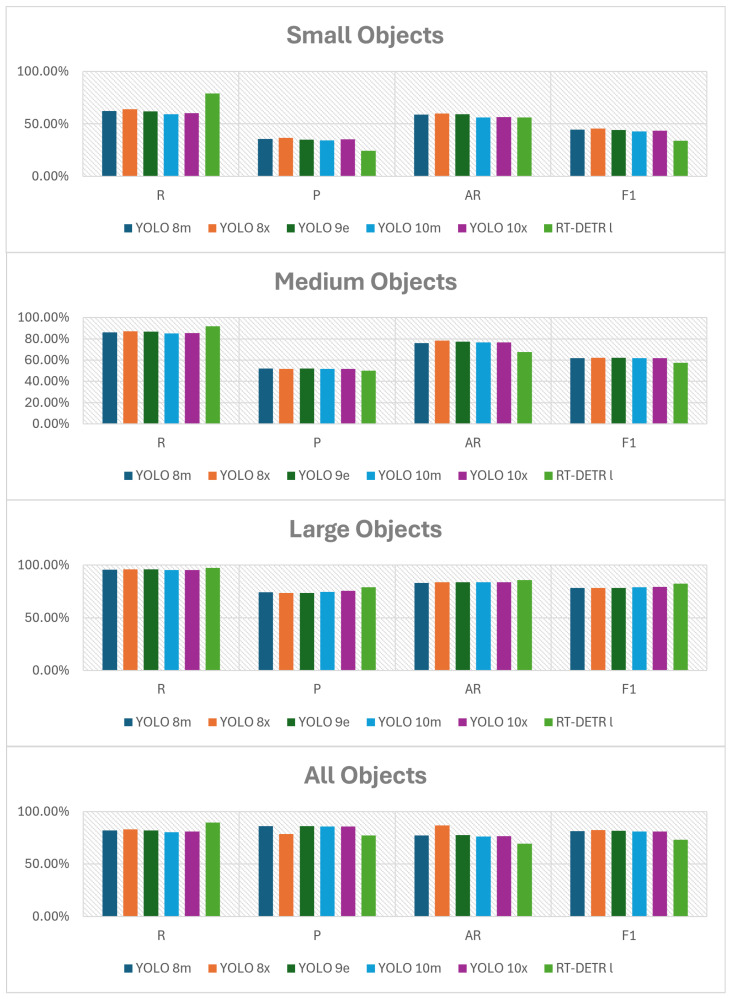
Comparison of P, R, F1-score, and AR metrics across different object sizes and models at optimal confidence thresholds.

**Figure 4:**
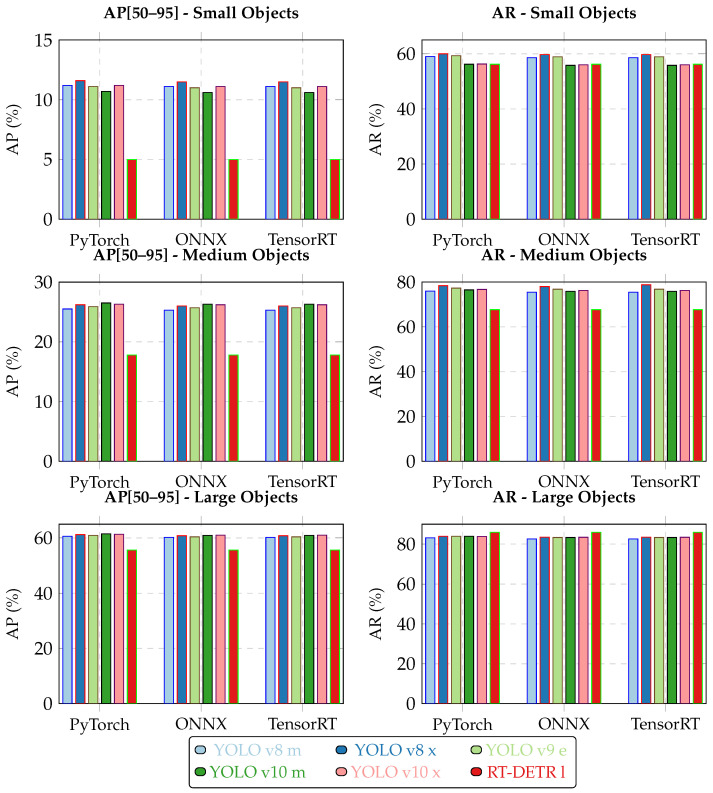
Comparison of AP[50–95] (**left** column) and AR at optimal confidence threshold (**right** column) across PyTorch, ONNX, and TensorRT formats for different object sizes. Each row represents a different object size category (Small, Medium, Large).

**Figure 5:**
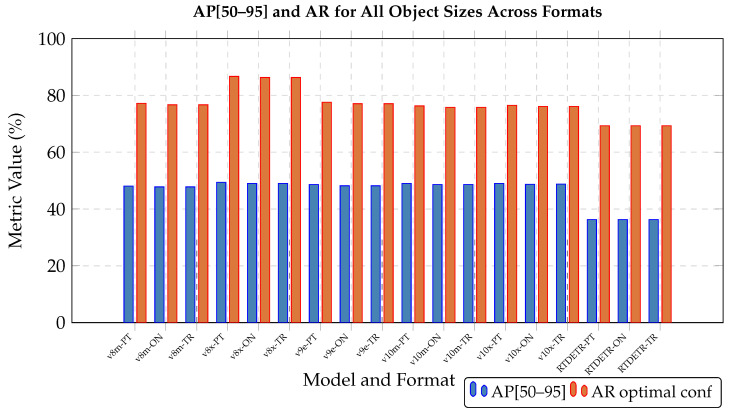
Comparison of AP[50–95] and AR metrics across all object sizes for PyTorch (PT), ONNX (ON), and TensorRT (TR) formats. Vertical dashed lines separate different model architectures.

The following figures summarize the performance of the evaluated object detection models. As shown in Figure 2 and Figure 3 (with the actual data provided in Table A1 and Table A2 in Appendix A), RT-DETR l performs worse than all YOLO variants. YOLO v10 x achieves the best results for small objects, YOLO v9 e performs best on medium objects, and YOLO v8 m leads for large bounding boxes. The accuracy differences between the PyTorch, ONNX, and TensorRT formats are presented in Figure 4 and Figure 5 (with the data available in Table A3 in Appendix A).

Statistical analysis across five training runs reveals several important findings. None of the pairwise comparisons showed statistically significant differences after Holm–Bonferroni correction, suggesting that the observed performance variations between models are within the range of random variation due to training stochasticity. The relatively small standard deviations (typically <2%) across runs, shown in Table 2, indicate that model training is reasonably stable despite the high heterogeneity of the meta-dataset.

Our experiments indicate that RT-DETR performs slightly worse than YOLO (v8/v9/v10) in detecting humans in infrared (IR) imagery. As shown in our results, RT-DETR L achieved only 36.3% AP[50–95] compared to 48.1–49.4% for YOLO variants, with particularly poor performance on small objects (5.0% AP[50–90] vs. 10.7–11.6% for YOLO models). Several factors may explain this performance gap.

#### 4.1.2. Dataset Characteristics and Scale Sensitivity

Human targets in thermal UAV imagery are typically very small—our dataset shows bounding boxes ranging from 4 px^2^ minimum to 40 px^2^ at the 10th percentile after resizing to 640-pixel width. RT-DETR’s transformer-based architecture with global attention may struggle with such extremely small, low-contrast thermal signatures that offer minimal textural information. The model achieved only 24.4% precision on small objects, compared to 36.7% for YOLO models, suggesting difficulty in learning discriminative features from sparse thermal footprints.

#### 4.1.3. Training Data Limitations

Our dataset, while extensive at 75,000+ images, combines heterogeneous sources with substantial domain shifts (urban vs. mountainous, varying altitudes, different sensor characteristics). Transformer-based detectors typically require larger and more consistent training datasets to effectively learn their attention mechanisms. The relatively limited size and high heterogeneity of thermal datasets compared to RGB benchmarks like COCO may disproportionately affect RT-DETR’s performance. This appears to be supported by our finding that the RT-DETR model achieved lower average precision than the YOLO models.

#### 4.1.4. Architectural Differences

YOLO’s CNN-based architecture with dense detection heads appears to be better suited for extracting local features from thermal blobs, which typically appear as bright, localized regions with minimal internal structure. The convolutional inductive bias may be more sample-efficient for this specific detection task. Additionally, RT-DETR’s set-based Hungarian matching approach may be less effective when dealing with numerous small, similar-looking thermal signatures that can be difficult to uniquely assign during training. Domain Transfer Issues: Both model families rely on pre-training from RGB datasets (COCO), creating a significant domain gap when applied to thermal imagery. However, our results suggest that this gap affects RT-DETR more severely. The 10–20× performance degradation of RT-DETR compared to YOLO variants indicates that the transformer architecture may be less robust to this particular domain shift, possibly because thermal images lack the rich texture and semantic context that transformers typically leverage in natural RGB images.

In summary, RT-DETR’s poor performance in our thermal human detection experiments appears primarily driven by the combination of extremely small target sizes, limited thermal-specific training data, and reduced robustness to the RGB-to-thermal domain gap. These findings suggest that CNN-based architectures like YOLO currently offer more reliable performance for thermal UAV-based search and rescue applications.

Format conversions introduced minor performance differences: ONNX and TensorRT generally showed slightly lower accuracy than PyTorch, though in some cases (e.g., RT-DETR), results were marginally better. These differences were small and consistent across model sizes. Due to poor accuracy, RT-DETR l was excluded from further evaluation. Model selection focused on YOLO variants. YOLO models were converted from PyTorch to ONNX and TensorRT, and they were evaluated on the Jetson AGX Orin under maximum power settings. Table 3 shows relative deviations, all within 1% of PyTorch accuracy. Performance results confirmed that PyTorch is not optimal for deployment. TensorRT offered the best balance of speed and efficiency, with negligible accuracy loss.

In addition to full-precision (TF32) evaluation, post-training quantization was explored to improve performance. Table 4 shows relative deviations for FP16 and INT8 compared to TF32, including energy and latency changes under Jetson AGX Orin’s MAXN power mode with the “jetson_clocks” script enabled. FP16 yielded substantial speed and energy gains—nearly halving power use—with a precision loss under 0.1%, making it a highly efficient alternative. In contrast, INT8 quantization showed 10–20% accuracy loss, making it less viable given the already modest baseline performance. These tradeoffs are discussed further in Section 4.2.1.

Our models achieve ~84–85% AP50 on the full meta-dataset (see Table 1), while AP[50–95] is substantially lower (~48%), reflecting the difficulty of small-object and cross-domain generalization; inference throughput on the Jetson ranges at ~3–15 FPS, depending on model and quantization.

#### 4.1.5. Semantic Segmentation

In this section, a brief presentation of the best results will be analyzed, deciding the method that yields the best performance for detecting people. The metric used to decide was the F1-score and F2-score, which both combine the precision and recall metrics.

**Figure 6:**
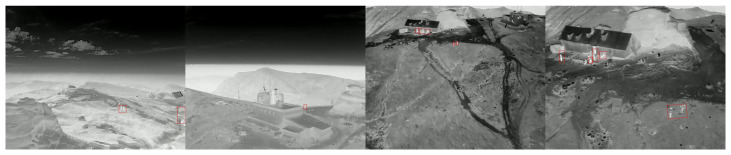
Example of Attention U-Net predictions on four difficult cases with small targets (in red bounding boxes) and high sky content.

Table 5 shows accuracy results obtained for the various segmentation methods we evaluated. These results indicate that the Attention U-Net is the most effective method for this specific task, utilizing a linear combination of Binary Cross-Entropy and Dice Loss. Figure 6 presents predictions on four challenging images, where the targets are small and more than half of the scene consists of irrelevant sky.

### 4.2. Embedded Performance

This section presents inference speed and energy consumption results—key metrics for real-time embedded systems. Tests were conducted on the Jetson AGX Orin Developer Kit (64GB RAM, Ampere GPU).

#### 4.2.1. Object Detection

**Figure 7:**
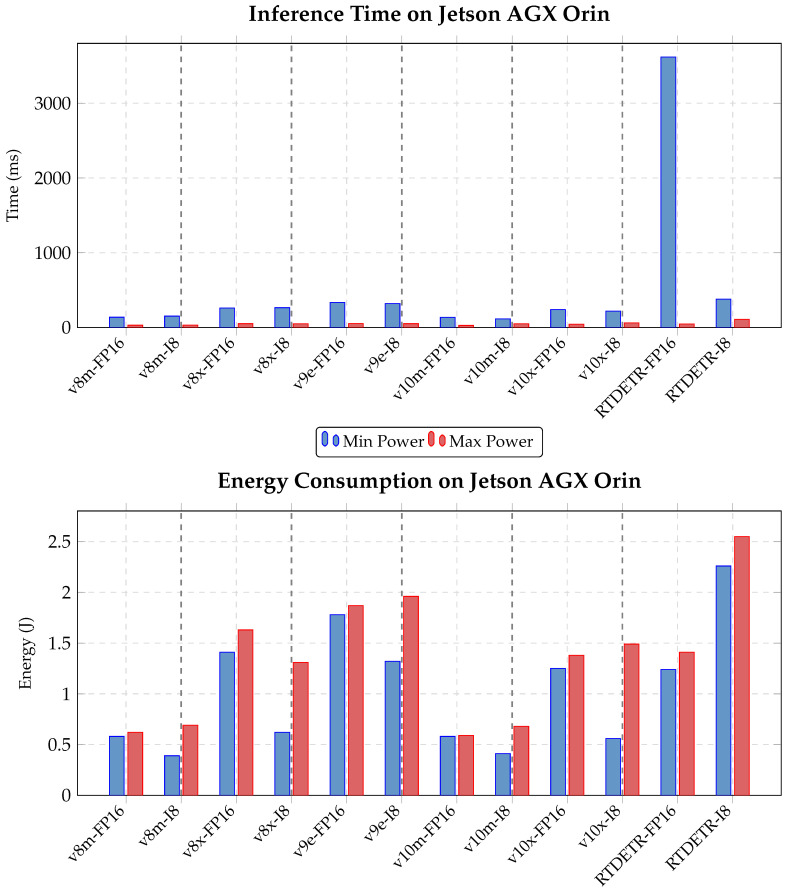
Inference time and energy consumption comparison for YOLO v8, v9, v10, and RT-DETR models on Jetson AGX Orin at minimum and maximum power modes. Each model is tested with both FP16 and INT8 (I8) precision. Lower is better.

Inference costs were evaluated over the full test set (15,033 images) under both power modes: 15W (Jetson clocks disabled) and MAXN (Jetson clocks enabled). Figure 7 reports the performance across all models (with data available in Table A4 in Appendix A). RT-DETR l showed the highest inference time and lowest accuracy, confirming its unsuitability for deployment.

**Figure 8:**
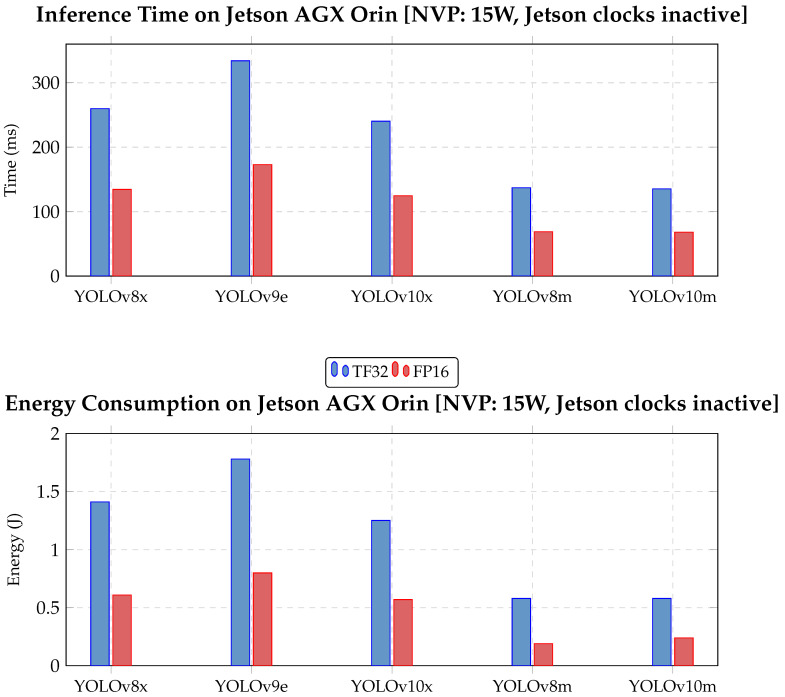
Inference time and energy consumption comparison for YOLO v8, v9, and v10 models on Jetson AGX Orin at 15W power mode. Each model is tested with TF32 and FP16 TensorRT precisions. Lower is better.

ONNX performed poorly in terms of processing speed and was excluded. While PyTorch and TensorRT were closer in performance, TensorRT was selected for offering the best tradeoff across power configurations. Post-training quantization was further evaluated for TensorRT. As noted in Section 4.1.1, INT8 caused excessive accuracy loss, so only the FP16 variant was retained. Figure 8 presents time and energy performance for YOLO models across TensorRT TF32, and TensorRT FP16 formats (with the data available in Table A5 in Appendix A).

The slowest YOLO model (YOLO v9 e, TensorRT FP16) achieves 5–6 FPS, which, while below a 25 FPS video stream rate, is still acceptable for real-time deployment, as not every frame must be processed. Overall, all models in FP16 are viable for real-time use on the Jetson AGX Orin, even under constrained conditions. YOLO v8 m offers the best energy efficiency, but YOLO v10 m delivers nearly identical performance with higher accuracy. Thus, YOLO v10 m in TensorRT FP16 represents the best overall tradeoff.

#### 4.2.2. Semantic Segmentation

Semantic segmentation inference was divided into three stages: pre-processing, inference, and post-processing. Pre-proc normalized input pixels to px∈[0,1]. Post-proc applied mask thresholding, dilation (for small objects), and bounding box generation. Processing time and power consumption for each stage is shown in Table 6.

### 4.3. Power Considerations

Results were consistent across both power modes: higher power allowed faster inference, while the minimal mode still supported real-time use. The power consumption of the board can be described as a function of the chosen inference rate. To save energy, one could consider reducing the FPS. This results in the global energy per image $E_{img}$ and average power $P_{avg}$ being calculated as $E_{img}=\frac{P_{avg}}{FPS}=\left(P_{idle}+t_{inf}\times E_{inf}\right)\times t_{inf}\;\mathrm{and}\;P_{avg}=P_{idle}+t_{inf}\times E_{inf}$, where $P_{avg}$ is the average power, FPS is the frame rate, $P_{idle}$ is the average idle power, $t_{inf}$ is the inference time, and $E_{inf}$ is the pure energy per inference. Finally, it is shown that an affine function can be used to describe the average power, or energy per second, required to power the Jetson during inference for a desired frame rate. Idle power consumption was estimated before each measurement. By averaging these values, we determined the Jetson AGX Orin’s idle power usage in each power mode. For the maximum power mode, $E_{inf}=0.28\;\mathrm{and}\;P_{idle}=11.41$

The domain of the function is $\left[0,FPS_{MAX}=50.62\right]$, which represents a power requirement ranging from 11.41 W (no inference) to 25.58 W (maximum inference speed). For the minimum power mode, $E_{inf}=0.24\;\mathrm{and}\;P_{idle}=6.39$.

The domain of the function is $\left[0,FPS_{MAX}=14.67\right]$, which represents a power requirement ranging from 6.39 W (no inference) to 9.91 W (maximum inference speed).

Power consumption of the Jetson AGX Orin increases with FPS, especially in max power mode, where 14.67 FPS uses 9.91 W in low power mode versus 15.53 W in high—over 30% more.

Power and energy measurements were obtained directly from the NVIDIA Jetson AGX Orin developer kit using the integrated “tegrastats” monitoring tool, which provides real-time readings of system power usage across the CPU, GPU, and memory domains. Each inference test was executed for a fixed number of frames to ensure stable average values, and idle power ($P_{idle}$) was recorded prior to each measurement to isolate the inference contribution. The inference energy ($E_{inf}$) was derived from the difference between the average measured power during inference and the idle baseline, multiplied by the inference duration per frame ($t_{inf}$). The proposed model assumes a linear (affine) relationship between power and frame rate, reflecting that each additional frame per second adds a nearly constant incremental energy cost. The proposed model assumes a linear relationship between power consumption and frame rate, based on the premise that each additional frame processed per second contributes a constant incremental energy cost. This assumption was adopted to simplify the estimation of average power and energy per image and was found to reasonably approximate the measured data across both power modes.

### 4.4. Comparison with State-of-the-Art

To contextualize our results within the broader literature, Table 7 compares our detection performance against recently published methods on overlapping benchmark datasets. We report mAP@50 where available, as this is the most commonly reported metric in thermal object detection literature.

Our single-dataset results (0.70–0.90 AP50) are competitive with or slightly below published SOTA on individual benchmarks, which is expected given that many recent methods employ dataset-specific architectural modifications (e.g., attention mechanisms tuned for specific object sizes or background characteristics). However, the critical finding is that while AP50 remains high (~0.85) for models trained on the full meta-dataset (Table 7), AP[50–95] is much lower (~0.44–0.49), indicating reduced performance across IoU thresholds and sensitivity to small objects and domain shifts.

## 5. Discussion

During our experiments, we observed that detection performance on the small objects subset remained relatively poor (approximately 28% AP50). This is consistent with the known issue that YOLO-style detectors are known to exhibit reduced performance on small objects due to the inherent downsampling in the backbone, grid-based assignment, and feature-pyramid design.

In terms of the models selected for this article, the main focus was on achieving an effective balance between detection accuracy and computational efficiency for deployment on embedded UAV platforms. U-Net architectures were chosen for their ability to perform precise pixel-level segmentation with relatively low computational requirements, making them suitable for onboard processing. Similarly, YOLO models were selected due to their proven real-time performance and streamlined inference pipeline, allowing rapid object detection with minimal latency. Models requiring high computational power were not considered, as their complexity and resource demands make them unsuitable for real-time inference on embedded systems with limited processing and energy capabilities.

## 6. Conclusions

This work addressed the challenge of reliable human detection in UAV thermal imagery, with a particular focus on mountain search and rescue scenarios where people often appear as small or partially occluded targets. We conducted a comparative study of state-of-the-art object detection and semantic segmentation methods with additional evaluation on an embedded platform. Training on the heterogeneous meta-dataset leads to a substantial drop in AP[50–95] (~48%) compared with single-dataset or environment-specific training. However, AP@50 remains high (~84%–85%) for the meta-trained YOLO models, while mountain-only training achieves AP@50 ~80% for the YOLOv11 exploratory test. Moreover, segmentation models proved more effective than detection-based approaches, with Attention U-Net reaching an F1-score of 0.91. On an NVIDIA Jetson AGX Orin, segmentation also achieved real-time performance, operating at up to 27 FPS in FP16 mode. These findings highlight two main contributions: (i) the introduction of a new mountain-oriented thermal image collection that complements existing resources, and (ii) a comprehensive evaluation of detection versus segmentation methods, including their embedded deployment characteristics. Together, they provide new insights into environment-specific training and model selection for UAV-based search and rescue.

## Figures and Tables

**Table 2:** Detection performance metrics across 5 training runs with different random seeds (7, 42, 999, 2024, 12,345). Values shown as mean ± standard deviation. Best results in bold.

Model	AP50 (%)	AP[50–95] (%)	Precision (%)	Recall (%)
yolov8m	84.13 ± 0.06	47.81 ± 0.04	85.81 ± 0.09	77.05 ± 0.12
yolov8x	**85.17 ± 0.14**	**49.10 ± 0.09**	**86.43 ± 0.22**	**78.40 ± 0.19**
yolov9e	84.39 ± 0.16	48.31 ± 0.08	85.95 ± 0.17	77.49 ± 0.18
yolov10m	84.15 ± 0.24	48.70 ± 0.22	85.61 ± 0.16	76.09 ± 0.30
yolov10x	84.45 ± 0.08	49.00 ± 0.05	85.89 ± 0.21	76.58 ± 0.20
rtdetr-l	74.98 ± 2.59	35.76 ± 1.27	76.24 ± 2.41	68.26 ± 2.21

**Table 3:** Summary of accuracy metrics and costs for each format of the custom YOLO models compared to the initial PyTorch format.

	**custom YOLO v8 x**		**custom YOLO v9 e**		**custom YOLO v10 x**	
Measure\Format	ONNX	TensorRT	ONNX	TensorRT	ONNX	TensorRT
AP[50–95]	49.0	49.0	48.2	48.1	48.7	48.8
AP Loss Relative to PyTorch	−0.72%	−0.72%	−0.83%	−0.85%	−0.55%	−0.52%
AR_Optimal Confidence_	86.3	86.3	77.1	77.0	76.1	76.2
AR Loss Relative to PyTorch	−0.49%	−0.49%	−0.69%	−0.71%	−0.51%	−0.49%
	**custom YOLO v8 m**		**custom YOLO v10 m**			
Measure\Format	ONNX	TensorRT	ONNX	TensorRT		
AP[50–95]	47.8	47.8	48.6	48.5		
AP Loss Relative to PyTorch	−0.65%	−0.65%	−0.91%	−0.92%		
AR_Optimal Confidence_	76.7	76.7	75.7	75.7		
AR Loss Relative to PyTorch	−0.65%	−0.65%	−0.75%	−0.75%		

**Table 4:** Summary of accuracy metrics and relative cost gains for different reduced-precision formats of the TensorRT custom YOLO models, evaluated on the NVIDIA Jetson AGX Orin (NVP mode: MAXN, Jetson clocks active).

	**custom YOLO v8 x**		**custom YOLO v9 e**		**custom YOLO v10 x**	
Measure\Format	TRT FP16	TRT INT8	TRT FP16	TRT INT8	TRT FP16	TRT INT8
AP[50–95]	49.3	44.9	48.6	35.5	48.9	44.5
AP Loss Relative to PyTorch	−0.06%	−8.55%	0.01%	−26.36%	−0.09%	−8.82%
AR_Optimal Confidence_	86.7	78.0	77.6	50.3	76.4	69.8
AR Loss Relative to TensorRT TF32	−0.04%	−10.82%	0.03%	−30.61%	−0.07%	−11.78%
Time Gain Relative to TensorRT TF32	40.48%	51.45%	37.19%	33.21%	34.97%	29.86%
Energy Difference Relative to TensorRT TF32	51.72%	75.34%	50.53%	94.79%	49.92%	72.74%
	**custom YOLO v8 m**		**custom YOLO v10 m**		N/A	
Measure\Format	TRT FP16	TRT INT8	TRT FP16	TRT INT8		
AP[50–95]	48.1	42.8	49.0	40.3		
AP Loss Relative to PyTorch	−0.03%	−13.43%	−0.03%	−18.90%		
AR_Optimal Confidence_	77.2	61.7	76.3	60.9		
AR Loss Relative to TensorRT TF32	0.00%	−21.07%	0.00%	−22.29%		
Time Gain Relative to TensorRT TF32	35.48%	40.03%	26.94%	−10.57%		
Energy Difference Relative to TensorRT TF32	53.38%	71.61%	51.90%	65.65%		

**Table 5:** Semantic segmentation aggregate results performance-wise. Best results in bold.

Method	Loss Function	Precision	Recall	F1-Score	F2-Score
U-Net	Basnet	0.9141	0.8705	0.8918	0.8789
U-Net++	Cross-Entropy	0.9068	0.8799	0.8931	0.8851
Residual U-Net	BCE Dice	0.9096	0.8802	0.8946	0.8859
Inception U-Net	Basnet	0.9194	0.8738	0.8960	0.8825
Dense U-Net	Basnet	0.9191	0.8811	0.8997	0.8884
**Attention U-Net**	**BCE Dice**	**0.9078**	**0.9185**	**0.9131**	**0.9163**
SE U-Net	Basnet	0.9072	0.8805	0.8937	0.8857

**Table 6:** Duration of inference and processing for the Attention U-Net (left). Power consumption and inference performance comparison under different power limitations (right).

Phase (ms)	GPU	CPU	Metric	No Lim	Max 15W
Pre-proc time	1.03	1.03	Energy Over Idle (avg)	0.70143 J	1.3213 J
Inference time	16.82	2080	Pre-processing Time (avg)	3.04 ms	7.21 ms
Post-proc time	3.2	3.2	Inference Time (avg)	33 ms	43 ms
Total time	21.06	2084	Post-processing Time	1.03 ms	2.43 ms
FPS	47.47	0.5	Average FPS	27	18.99

**Table 7:** Comparison with state-of-the-art methods on public thermal datasets. Our results are from models trained on individual dataset subsets (not the full meta-dataset) to ensure fair comparison.

Dataset	Method	mAP50	Reference
FLIR ADAS	LFIR-YOLO	0.72	Wang et al., 2024 [71]
FLIR ADAS	YOLOv9t	0.72	Wang et al., 2024 [71]
FLIR ADAS	Our YOLOv10 (subset)	0.70	This work
LLVIP	LI-YOLO	0.90	Liu et al., 2024 [72]
LLVIP	YOLOv8	0.90	Liu et al., 2024 [72]
LLVIP	Our YOLOv10 (subset)	0.85	This work
HIT-UAV	YOLO-ViT	0.93	Zhao et al., 2023 [73]
HIT-UAV	YOLOv7s	0.93	Suo et al., 2023 [53]
HIT-UAV	Our YOLOv10 (subset)	0.88	This work
BIRDSAI	MBAF-MSC	0.93	Wu et al., 2025 [74]
BIRDSAI	Our YOLOv10 (subset)	0.89	This work
RGBTDronePerson	VTSaR-Trans	0.94	Zhang et al., 2025 [75]
RGBTDronePerson	YOLOv5s	0.94	Zhang et al., 2023 [32]
RGBTDronePerson	Our YOLOv10 (subset)	0.90	This work
Meta-dataset (ours)	Our YOLOv9e	0.85	This work
Mountain (ours)	Our YOLOv11	0.80	This work

## Data Availability

The data presented in this study are available on request from the corresponding author due to human detection is a sensitive application. In order to request access to the image dataset, please fill in the form at https://arh.dcae.pub.ro/projects/people-detection-systems-for-uav/thermal-aerial-images-database-for-people-detection/ (accessed on 25 August 2025).

## Abbreviations

The following abbreviations are used in this manuscript:

UAV	Unmanned Aerial Vehicle
SAR	Search and Rescue
GPU	Graphical Processing Unit
YOLO	You Only Look Once
CNNs	Convolutional Neural Networks
ADAS	Advanced Driver-Assistance System
TIR	Thermal Infrared
RGB	Red Green Blue

## Appendix A

This Annex provides the data in numerical format that was used for the charts in Figure 2, Figure 3, Figure 4, Figure 5, Figure 6, Figure 7 and Figure 8.

**Table A1:** Values of the metrics AP50, AP75, AP[50–95] for the custom-trained models at their considered optimal confidence threshold, based on the size of the bounding boxes. Best values are in bold.

Size/Model	YOLO v8 m			YOLO v8 x			YOLO v9 e		
	AP50	AP75	AP[50–95]	AP50	AP75	AP[50–95]	AP50	AP75	AP[50–95]
Small	27.8	6.9	11.2	**28.6**	**7.1**	**11.6**	27.3	6.7	11.1
Medium	50.3	22.3	25.5	51.0	23.4	26.2	50.7	23.0	25.9
Large	85.2	69.8	60.6	85.2	70.6	61.2	85.5	70.3	60.9
All	84.3	47.7	48.1	**85.4**	**49.3**	**49.4**	84.6	48.4	48.6
Size/Model	YOLO v10 m			YOLO v10 x			RT-DETR l		
	AP50	AP75	AP[50–95]	AP50	AP75	AP[50–95]	AP50	AP75	AP[50–95]
Small	26.7	6.3	10.7	27.3	7.0	11.2	18.3	0.9	5.0
Medium	**51.1**	**23.9**	**26.5**	50.8	23.8	26.3	46.1	9.0	17.8
Large	85.9	**71.2**	**61.6**	86.1	70.7	61.3	**89.4**	62.2	55.6
All	84.3	49.2	49.0	84.4	**49.3**	49.0	76.7	29.8	36.3

**Table A2:** Values of the metrics P, R, F1-score, and AR for the custom-trained detection models at their considered optimal confidence threshold, based on the size of the bounding boxes. Best values are in bold.

Size/Model	YOLO v8 m				YOLO v8 x			
	P	R	F1-Score	AR	P	R	F1-Score	AR
Small	35.6	62.3	44.4	59.0	**36.7**	63.9	**45.5**	**60.0**
Medium	52.1	86.0	61.8	76.0	51.7	87.2	**62.3**	**78.4**
Large	74.3	95.8	78.5	83.1	73.7	96.1	78.5	83.9
All sizes	85.9	81.8	81.3	77.2	78.5	82.9	**82.4**	**86.7**
Size/Model	YOLO v9 e				YOLO v10 m			
	P	R	F1-Score	AR	P	R	F1-Score	AR
Small	35.1	61.9	44.1	59.3	34.5	59.1	42.8	56.2
Medium	**52.2**	86.8	**62.3**	77.3	51.9	85.0	61.9	76.5
Large	73.5	96.0	78.4	83.9	74.8	95.3	79.1	83.9
All sizes	**86.1**	82.1	81.6	77.6	85.8	80.3	80.8	76.3
Size/Model	YOLO v10 x				RT-DETR l			
	P	R	F1-Score	AR	P	R	F1-Score	AR
Small	35.5	60.1	43.5	56.3	24.4	**79.0**	34.0	56.2
Medium	51.6	85.3	61.7	76.7	50.0	**91.8**	57.5	67.7
Large	75.5	95.4	79.4	83.8	**79.0**	**97.4**	**82.3**	**85.9**
All sizes	85.8	80.8	80.9	76.5	77.1	**89.6**	73.0	69.3

**Table A3:** Values of AP[50–95] and AR at the optimal confidence threshold for each model and each category of bounding box sizes for the PyTorch, ONNX, and TensorRT formats.

	YOLO v8 m						YOLO v8 x					
	PyTorch		ONNX		TensorRT		PyTorch		ONNX		TensorRT	
Size\Metric	AP [50–95]	AR optimal conf	AP [50–95]	AR optimal conf	AP [50–95]	AR optimal conf	AP [50–95]	AR optimal conf	AP [50–95]	AR optimal conf	AP [50–95]	AR optimal conf
Small	11.2	59.0	11.1	58.6	11.1	58.6	11.6	60.0	11.5	59.7	11.5	59.7
Medium	25.5	76.0	25.3	75.5	25.3	75.5	26.3	78.4	26.0	78.0	26.0	78.0
Large	60.6	83.1	60.2	82.6	60.2	82.6	61.2	83.9	60.8	83.5	60.8	83.5
All sizes	48.1	77.2	47.8	76.7	47.8	76.7	49.4	86.7	49.0	86.3	49.0	86.3
	YOLO v10 m						YOLO v10 x					
	PyTorch		ONNX		TensorRT		PyTorch		ONNX		TensorRT	
Size\Metric	AP [50–95]	AR optimal conf	AP [50–95]	AR optimal conf	AP [50–95]	AR optimal conf	AP [50–95]	AR optimal conf	AP [50–95]	AR optimal conf	AP [50–95]	AR optimal conf
Small	10.7	56.2	10.6	55.8	10.6	55.8	11.2	56.3	11.1	56.0	11.1	56.0
Medium	26.5	76.5	26.3	75.9	26.3	75.9	26.3	76.7	26.12	76.3	26.2	76.3
Large	61.5	83.9	60.9	83.3	60.9	83.3	61.3	83.8	61.0	83.4	61.0	83.4
All sizes	49.0	76.3	48.6	75.7	48.6	75.7	49.0	76.5	48.7	76.1	48.8	76.1
	YOLO v9 e						RT-DETR l					
	PyTorch		ONNX		TensorRT		PyTorch		ONNX		TensorRT	
Size\Metric	AP [50–95]	AR optimal conf	AP [50–95]	AR optimal conf	AP [50–95]	AR optimal conf	AP [50–95]	AR optimal conf	AP [50–95]	AR optimal conf	AP [50–95]	AR optimal conf
Small	11.1	59.3	11.0	58.9	11.0	58.9	5.0	56.2	5.0	56.2	5.0	56.2
Medium	25.9	77.3	25.7	76.8	25.7	76.8	17.8	67.7	17.8	67.7	17.8	67.7
Large	60.9	83.9	60.4	83.3	60.4	83.3	55.6	85.9	55.6	85.9	55.6	85.9
All sizes	48.6	77.6	48.2	77.0	48.2	77.0	36.3	69.3	36.3	69.3	36.3	69.3

**Table A4:** Inference costs for YOLO v8, v9, v10 and RT-DETR on Jetson AGX Orin.

Power	Metric	v8 m		v8 x		v9 e		v10 m		v10 x		RT-DETR	
		FP16	I8	FP16	I8	FP16	I8	FP16	I8	FP16	I8	FP16	I8
Min	Time (ms)	137	152	260	265	334	319	135	115	240	218	3618	379
	Energy (J)	0.58	0.39	1.41	0.62	1.78	1.32	0.58	0.41	1.25	0.56	1.24	2.26
Max	Time (ms)	30	31	51	49	53	51	27	49	43	62	46	109
	Energy (J)	0.62	0.69	1.63	1.31	1.87	1.96	0.59	0.68	1.38	1.49	1.41	2.55

**Table A5:** Performance of custom YOLO models for TensorRT TF32, and TensorRT FP16 formats, obtained on the NVIDIA Jetson AGX Orin [NVP: 15W, Jetson clocks inactive].

	YOLO v8 x		YOLO v9 e		YOLO v10 x		YOLO v8 m		YOLO v10 m	
Metric/Format	TF32	FP16	TF32	FP16	TF32	FP16	TF32	FP16	TF32	FP16
Inference time (ms)	259.82	134.40	334.10	172.84	240.33	124.68	136.98	68.68	135.33	68.17
FPS	3.85	7.44	2.99	5.79	4.16	8.02	7.30	14.56	7.39	14.67
Energy/img (J)	1.41	0.61	1.78	0.80	1.25	0.57	0.58	0.19	0.58	0.24
